# A pharmacist-led opioid de-escalation program after completion of chemoradiotherapy in locally advanced head and neck cancer

**DOI:** 10.3389/fonc.2023.1145323

**Published:** 2023-09-15

**Authors:** Ai Horinouchi, Tomohiro Enokida, Shinya Suzuki, Hayato Kamata, Asumi Kaneko, Chihiro Matsuyama, Takao Fujisawa, Yuri Ueda, Kazue Ito, Susumu Okano, Toshikatsu Kawasaki, Makoto Tahara

**Affiliations:** ^1^ Department of Pharmacy, National Cancer Center Hospital East, Kashiwa, Japan; ^2^ Department of Pharmacy, South Miyagi Medical Center, Ōgawara, Japan; ^3^ Department of Head and Neck Medical Oncology, National Cancer Center Hospital East, Kashiwa, Japan; ^4^ Department of Clinical Pharmacology and Therapeutics, Kyoto University Hospital, Kyoto, Japan; ^5^ Department of Otorhinolaryngology-Head and Neck Surgery, Tokyo Medical University Hospital, Shinjuku-ku, Japan; ^6^ Department of Head and Neck Medical Oncology, Miyagi Cancer Center, Natori, Japan

**Keywords:** head and neck cancer, opioid, tapering, chemoradiation therapy, oral mucositis

## Abstract

**Background:**

Persistent opioid use frequently leads to substantial negative impacts on quality of life, and as the outlook for numerous cancer types continues to improve, these complications become increasingly crucial. It is essential to acknowledge that extended or excessive opioid use may result in adverse effects in patients who completed radiation therapy (RT).

**Methods:**

In this time-series analysis, we compared the outcomes of patients who participated in the pharmacist-led opioid de-escalation (PLODE) program after completing concurrent radiotherapy (CRT) between June 2018 and February 2019 against patients who completed CRT between June 2017 and March 2018 and did not participate in the program.

**Results:**

Among 61 patients, 16 (26%) used opioids after completing CRT and participated in the PLODE program. Before starting the program, 93 patients completed CRT between June 2017 and March 2018 and 32 (34%) used opioids at CRT completion. These patients were deemed the control group. In the PLODE group, outpatient pharmacist intervention was performed, with 29 total interventions related to opioid use, of which 16 (55%) recommended tapering or discontinuing opioids according to the definition of this program. Patients who participated in the PLODE program discontinued opioids significantly earlier than those in the control group (median time to opioid discontinuation 11 days vs. 24.5 days, *p* < 0.001). None of the patients in the PLODE group resumed opioid use following discontinuation or escalated opioid dosing due to worsening pain.

**Conclusion:**

This study showed the utility of pharmacist-initiated interventions for opioid use in patients with head and neck cancer who had completed CRT.

## 1 Introduction

Surgery and radiation therapy are definitive treatments for local squamous cell carcinoma of the head and neck (SCCHN), and concurrent platinum-based chemoradiotherapy has been used as the standard of care in both definitive and adjuvant settings (1–5). One of the most common and debilitating toxicities of the treatment is radiation-induced mucositis due to several factors, including DNA damage caused by reactive oxygen generated by radiation and chemotherapy, and a bacterial infection caused by reduced local immune function (6–8) Reportedly, the incidence of oral mucositis in the population ranges from 50 to 90%, depending on the radiotherapy field, dose, fractionation, and chemotherapy administration (9). Notably, it has been reported that radiation-induced mucositis can be the main reason for unplanned breaks in radiotherapy. Thus, management of oral mucositis in head and neck cancer (HNC) patients treated with concurrent radiotherapy (CRT) is a critical issue as it comprises one of the most common complications leading to unexpected treatment interruption as well as hospitalization, associated with a remarkably worse prognosis (10–15).

Efforts were made to reduce mucositis through supportive therapies such as the oral care program reported by Yokota et al., as well as the implementation of cryotherapy, mucosal protective agents, and lidocaine preparations advocated by the NCCN (11, 14). Regarding the use of analgesics, Acetaminophen is primarily used for mild pain, while opioids are used for moderate to severe pain to achieve a high CRT completion rate while managing pain (12, 13). At our hospital, the initial response for all patients undergoing radiation therapy involves providing oral care utilizing Azunol mouthwash and lidocaine-containing mouthwash. If the mucositis-related pain worsens, acetaminophen or opioids are administered based on the pain level, following the protocol outlined in a previously reported opioid-based pain control program (12). However, the pain caused by CRT usually gradually disappears after the completion of treatment. A phase 2 study, which investigated an oral care program for radiation-induced oral mucositis (functional/symptomatic), reported that grade 3 oral mucositis was observed in 24.8% and 6.3% at two weeks and four weeks after CRT, respectively (14). In these circumstances, irresponsible opioid administration without consideration of the dynamic course can cause opioid-related adverse events that are detrimental to the patient. Currently, it is not feasible to engage in discussions regarding opioid taper or discontinuation with physicians, even if pharmacists are responsible for managing opioids in the outpatient setting after CRT. This is primarily due to the lack of evidence or relevant previous studies that can offer guidance on the appropriate timing for tapering opioids. Consequently, the medication is maintained at the same dosage.

Herein, we report the potential value of a pharmacist-led opioid de-escalation program in patients with locally advanced SCCHN who had completed chemoradiotherapy and used opioids to manage the treatment-related pain with a focus on opioid tapering in order to avoid opioid overdosing in the population.

## 2 Materials and methods

### 2.1 Study design and subjects

This time-series analysis compared the outcomes of patients who participated in the pharmacist-led opioid de-escalation (PLODE) program, in which outpatient pharmacists assisted decision-making regarding opioid tapering in collaboration with the medical oncologist after completing CRT or bioradiotherapy (BRT), against those who did not participate in the program in the same clinical settings, in order to evaluate the usefulness of the program. All subjects in this study were treated at the National Cancer Center Hospital East (NCCHE) and used opioids for pain due to treatment-related mucositis. Patients who participated in the de-escalation program between June 2018 and February 2019 were classified into the PLODE group, and those who did not participate in the program between June 2017 and March 2018 were used as historical controls. We retrospectively reviewed their medical records regarding the duration of opioid use and the clinical course after radiation.

The exclusion criteria to extract the population included patients who (1) participated in a clinical trial, (2) used opioids other than mucositis due to RT, (3) stopped using opioids before the completion of RT, (4) were treated with proton beam therapy, and (5) received a single dose of cisplatin during CRT.

In this study, the sample size encompassed all eligible patients during the specified case collection period, and there was no patient overlap observed between the PLODE and control groups.

### 2.2 PLODE program

The services provided by clinical pharmacists collaborating with oncologists were divided into three categories based on time: (1) before the oncologist outpatient examination, (2) during the oncologist outpatient examination, (3) and after the oncologist outpatient examination.

**Figure 1:**
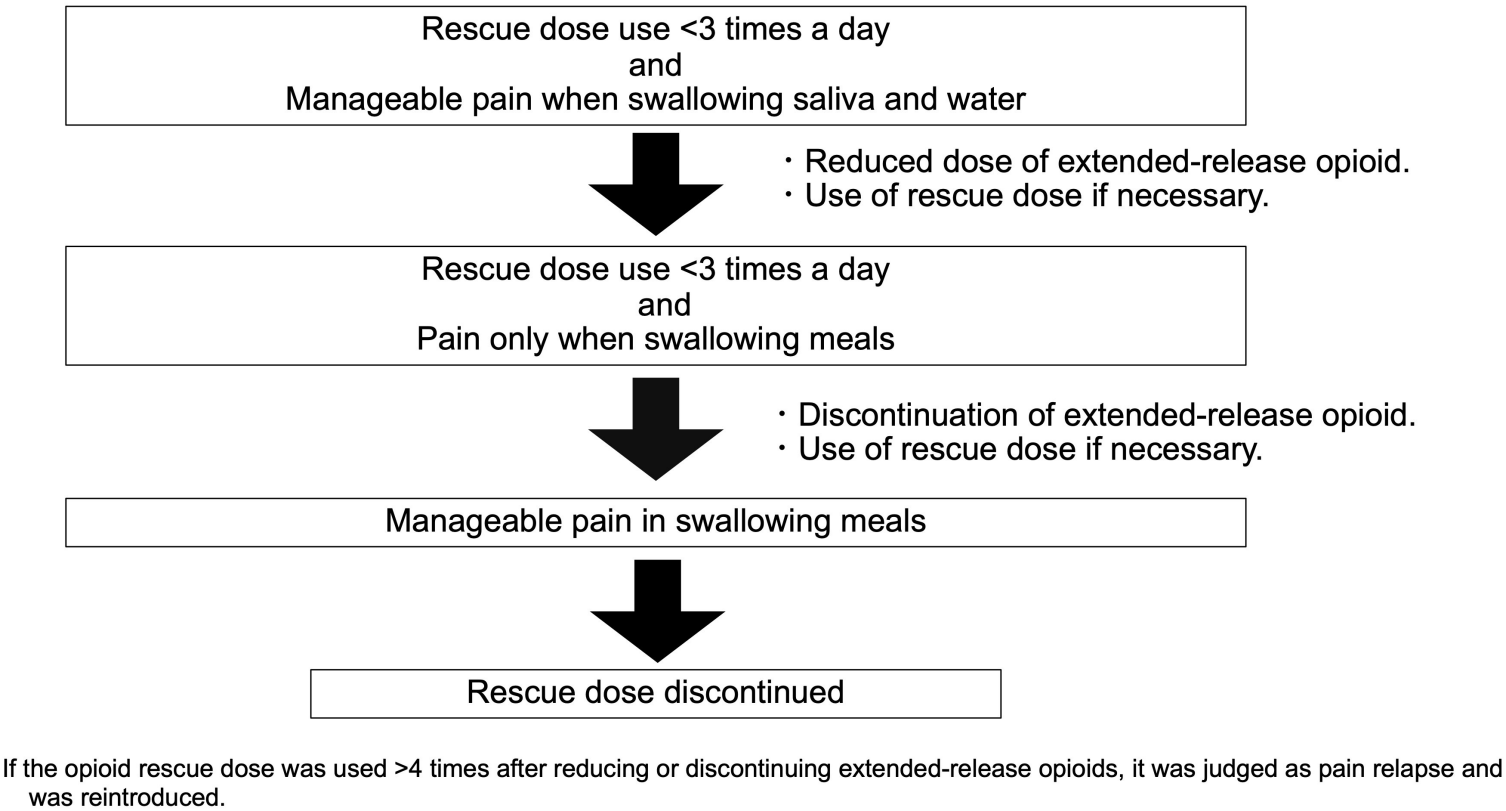
Protocol of the pharmacist-led opioid de-escalation (PLODE) program.

In the PLODE program, the outpatient pharmacist checked (1) the number of short-acting opioid rescues, (2) complaints of pain before and after opioid tapering, and (3) the purpose of opioid use, such as pain when swallowing meals or at other times on the day of clinic visit before the doctor visit, according to the flow chart (**Figure 1**). If the pharmacist judged that the pain had improved enough to taper the opioid, they suggested opioid tapering during the doctor’s visit. The doctor thoroughly reviewed the pain-related information provided by the pharmacist and the patient’s actual symptoms before reaching a final decision. In cases where opioid tapering was implemented, the pharmacist instructed the patient to use an opioid rescue option when the pain recurred as a result of the tapering process. Since the Palliative Medicine Society, based on ESMO guidelines, recommends that the use of opioid rescue doses more than four times may require an increase in the regular dose of opioids (16), we considered three or fewer uses of rescue doses as a sign of tapering opioid use in this program. Briefly, (1) if a rescue dose was used less than three times a day, and the pain was manageable even while swallowing saliva and water, a reduction in the extended-release opioid dose was suggested against using the rescue dose; (2) when patients were administered the minimum dose of extended-release opioids and the rescue dose was used less than three times a day, and the pain was observed only when swallowing meals, discontinuation of extended-release opioids under a free use of rescue dose was suggested; and (3) in cases where patients used only opioid rescue doses with the pain being acceptable while swallowing, discontinuation of rescue dose opioid and complete switching to non-opioid pain management if necessary was suggested. At any stage, if the opioid rescue dose was used more than four times after reducing or discontinuing an extended-release opioid, it was considered a pain relapse and the extended-release opioid could be reintroduced.

### 2.3 Data analysis

In the PLODE group, the duration of opioid use was defined from the end of radiation to the last day of opioid use, which was confirmed based on records maintained by the patient. In the control group, the last day of opioid use was the day after the last prescription day if the patient had only used a short-acting opioid. In case an extended-release opioid was used, the last prescription day of the opioid was defined as the last opioid use date for the particular subject.

Along the course of treatment, the maximum daily dose of opioid use, dose of acetaminophen used at the initiation of opioid use, the incidence of grade 3 mucositis (symptom/function), the radiation dose at the onset of mucositis, the maximum grade and the initiation of opioid use, the duration from the start of radiation to opioid use, duration of opioid use after completion of radiation, and total duration of administration were investigated. In addition, opioid-related adverse events such as nausea/vomiting, constipation, and sleepiness at the start of the PLODE program were also investigated. Oral mucositis and opioid-related adverse events were evaluated using the Common Terminology Criteria for Adverse Events (CTCAE) ver. 4.0.

### 2.4 Interventions for opioid use by a pharmacist

Pharmacist-initiated interventions referred to actions such as interviews with the patient who used opioids by pharmacists before the oncologist’s outpatient examination to confirm the occurrence of opioid-induced side effects, pain levels, medication compliance, and suggest a prescription. Pharmacists’ suggestions to physicians regarding opioid prescriptions were defined as prescribing interventions.

### 2.5 Statistics

Fisher’s exact probability test was used for categorical variables, whereas the Mann-Whitney U-test was used to compare the period from the start or completion of radiation to the occurrence of each event between the two groups. All statistical analyses were performed using SPSS software (version 17.00, SPSS, Inc., Chicago, IL, USA) for the statistical analysis. *S*tatistical significance was set at *p* < 0.05. In this study, a *post-hoc* power analysis was used to calculate the *post-hoc* power and to evaluate the sample size.

### 2.6 Ethics approval

This study was approved by the National Cancer Institutional Review Board (approval #2018-362). Since this was a retrospective study, the requirement for informed consent was waived.

## 3 Results

### 3.1 Patients characteristics

Sixty-one patients underwent CRT between June 2018 and February 2019. Among these, 16 (26%) used opioids at the time of CRT completion and participated in the PLODE program. Since 93 patients completed CRT between June 2017 and March 2018 and 32 (34%) used opioids at CRT completion, these patients were deemed the control group. At our hospital, a gastrostomy is performed for all patients before starting CRT, as radiation therapy can worsen oral mucositis and hinder oral medication administration. Therefore, in all cases, morphine granules were used for extended-release opioid doses, and oral morphine solution was utilized for rescue doses. These options are chosen because they can be easily administered through the gastrostomy. Most of the chemotherapy combined with radiation was cisplatin. Patient characteristics are shown in **Table 1**. There was a remarkable difference between the two groups only in the percentage distribution of clinical stages but not in stage 4 proportions. Other backgrounds were similar between the two groups. During the study period, all patients in the PLODE and control groups demonstrated no residual or recurrent tumors following CRT.

**Table 1:** Patient characteristics.

Characteristic	No. of patients (%)		*p*-value^†^
	PLODE (n=16)	Control (n=32)	
**Gender**			0.72
Male	13 (81)	23 (72)	
Female	3 (19)	9 (28)	
**Age median** years [range]	64 [31–73]	60 [32–75]	0.35
**Primary site**			0.58
Nasopharynx	1 (6)	4 (12)	
Oropharynx	6 (38)	16 (50)	
Hypopharynx	3 (19)	6 (19)	
Oral cavity	4 (25)	5 (16)	
Larynx	1 (6)	1 (3)	
Unknown primary	1 (6)	0 (0)	
**Stage (UICC 8th edit)**			0.0006
I	3 (19)	9 (28)	
II	3 (19)	0 (0)	
III	2 (13)	10 (31)	
IV	8 (50)	13 (40)	
**Clinical setting**			0.28
Definitive	12 (75)	28 (88)	
Postoperative	4 (25)	4 (12)	
**Treatment strategy**			0.66
IC→CRT	6 (38)	10 (31)	
CRT/BRT	10 (62)	22 (69)	
**Combination chemotherapy**			0.15
CDDP	14 (88)	32 (100)	
Cmab	1 (6)	0 (0)	
CBDCA	1 (6)	0 (0)	
CBDCA+5-FU	0 (0)	0 (0)	
**Median radiation doses**, Gy [range]	66 [66–70]	66 [64–70]	0.72
**Median radiation dose at the onset of mucositis**, Gy [range]	24 [14–36.04]	23 [13–48.56]	0.62
**Median radiation dose at the maximum grade of mucositis**, Gy [range]	54 [34–70]	58 [13–70]	0.93
**Incidence of grade 3 oral mucositis at the completion of RT**	11 (68)	23 (72)	0.82
**Median radiation dose at the onset of mucositis**, Gy [range]	24 [14–36.04]	23 [13–48.56]	0.31
**Median radiation dose at the maximum grade**, Gy [range]	54 [34–70]	58 [13–70]	0.46
**Median radiation dose at the start of opioid use**, Gy [range]	45 [22–69.96]	36 [16–63.6]	0.17
**Average maximum dose of opioid use at the completion of RT ± SD**, mg/day*	27 ± 15	30 ± 18	0.39
**Average dose of acetaminophen used at the completion of RT ± SD**, mg/day	1,818 ± 811	2,115 ± 752	0.21
**Median duration from the start of radiation to opioid use**, days [range]	32 [14–50]	24 [9–46]	0.12

CBDCA, carboplatin; CDDP, cisplatin; Cmab, cetuximab; CRT, chemoradiation therapy; IC, induction chemotherapy; 5-FU, 5-fluorouracil; SD, standard deviation. ^*^ Oral morphine equivalent dose. ^†^p-values were determined using the χ^2^ test and Mann–Whitney U test.

### 3.2 Side effects of opioids observed at the initiation of the PLODE program

**Figure 2:**
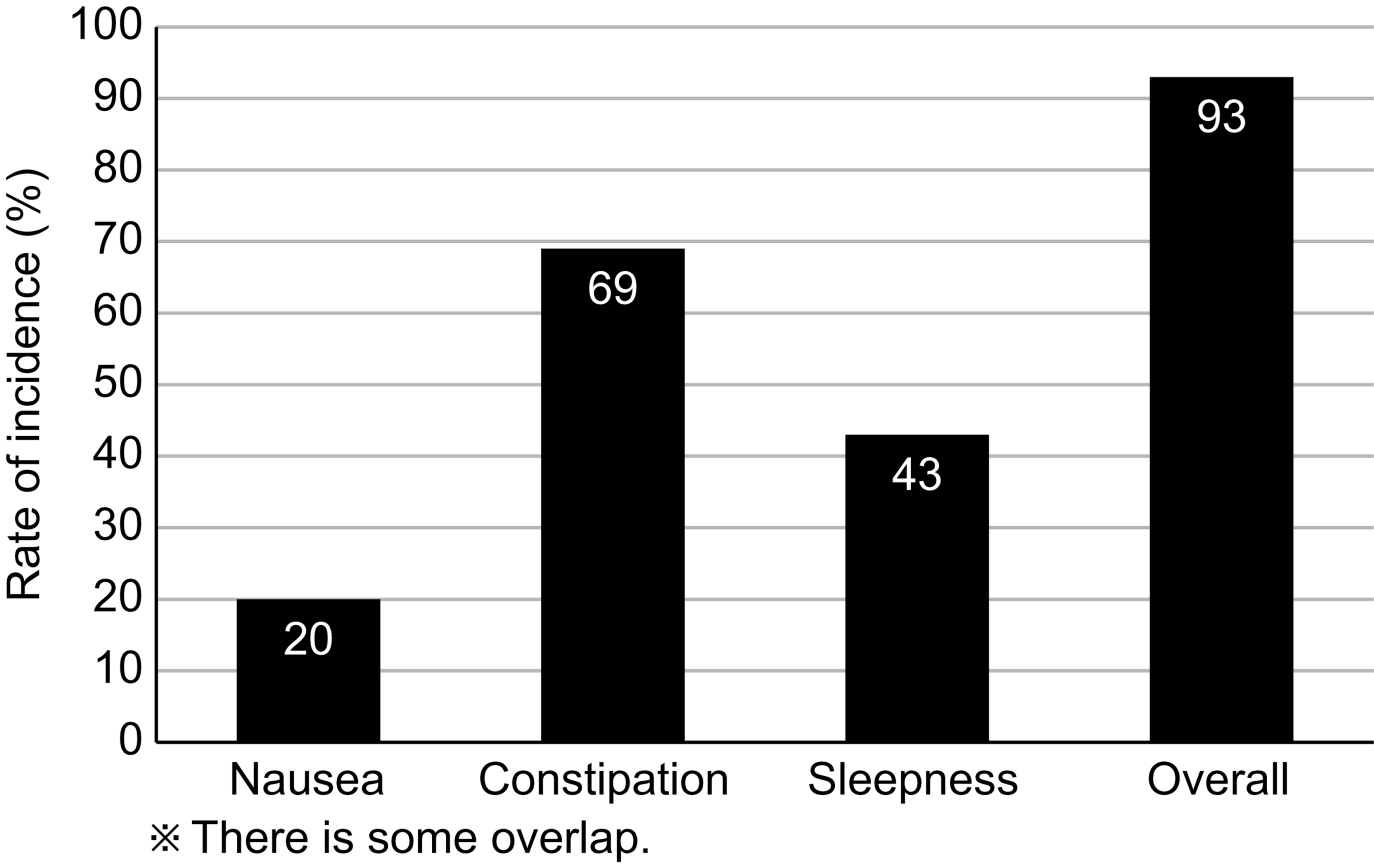
Opioid-induced side effects (All Grade) observed at the initiation of PLODE program.

**Figure 2** shows the opioid-induced side effects at the initiation of the PLODE program, which were subsequently coped by pharmacists in the PLODE group. The overall incidence of adverse effects was 93%, and the incidence of nausea, constipation, and sleepiness was 20%, 69%, and 43%, respectively. Unfortunately, in the control group, only the side effects documented in the medical record could be verified in the retrospective survey. Nonetheless, the overall incidence of adverse effects was 65%, and the incidences of nausea, constipation, and sleepiness were 62%, 57%, and 29%, respectively.

### 3.3 Pharmacist interventions for opioid use

**Figure 3:**
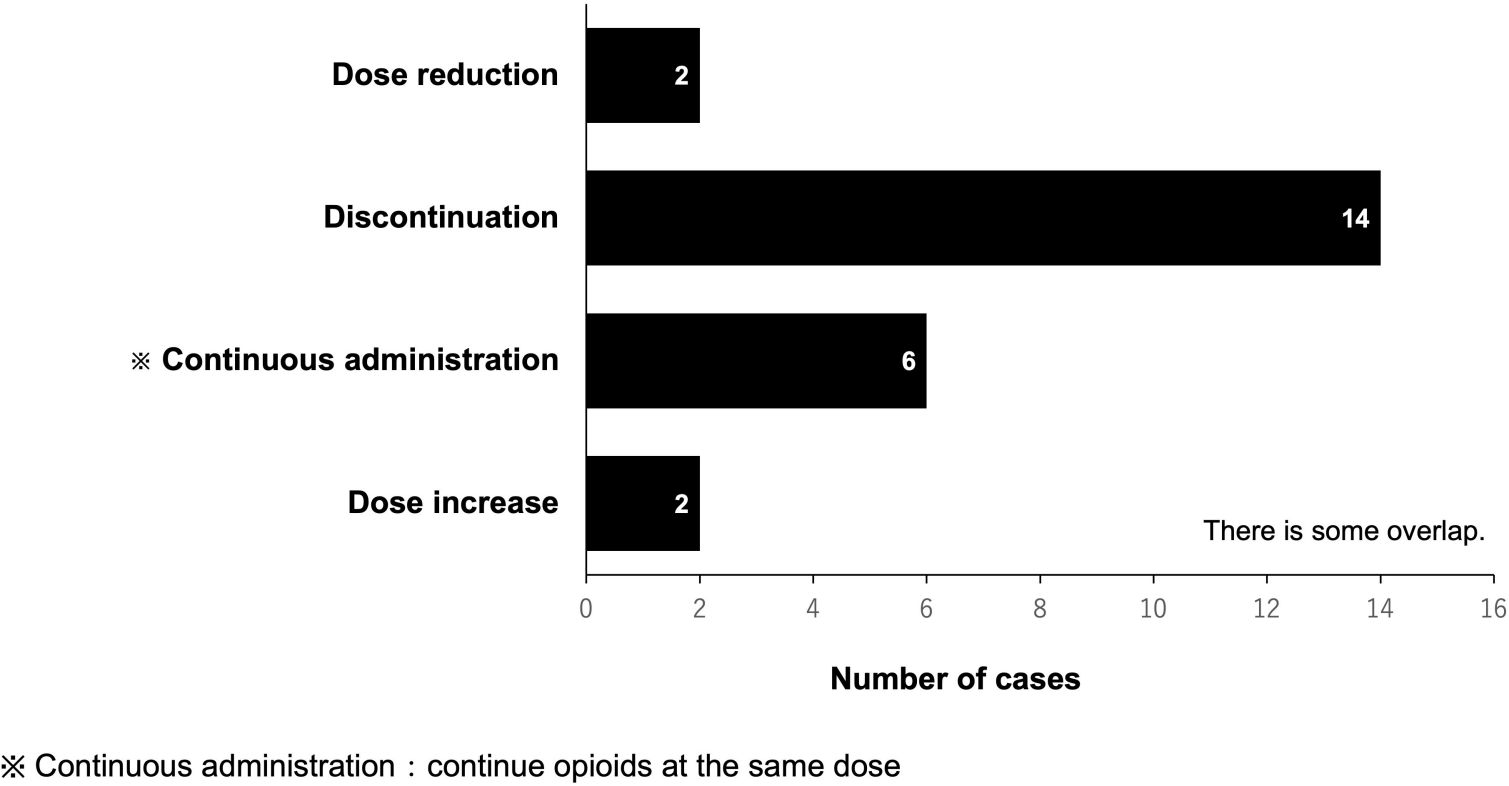
Pharmacist-led interventions for opioid use after CRT.

In the PLODE group, the total number of pharmacist-led interventions for opioid use during and after CRT was 57 (16 patients). Among them, the total number of prescriptions proposed by pharmacists was 24, of which the physician accepted 22 prescriptions (91%) in 15 patients with a median acceptance of one time/patient. The most common pharmacist’s suggestion was to discontinue opioids (14/24 times, 58.3%). However, there were only two suggestions for opioid dose increase (**Figure 3**).

### 3.4 Change in the number of patients using opioids after completion of radiotherapy

**Figure 4:**
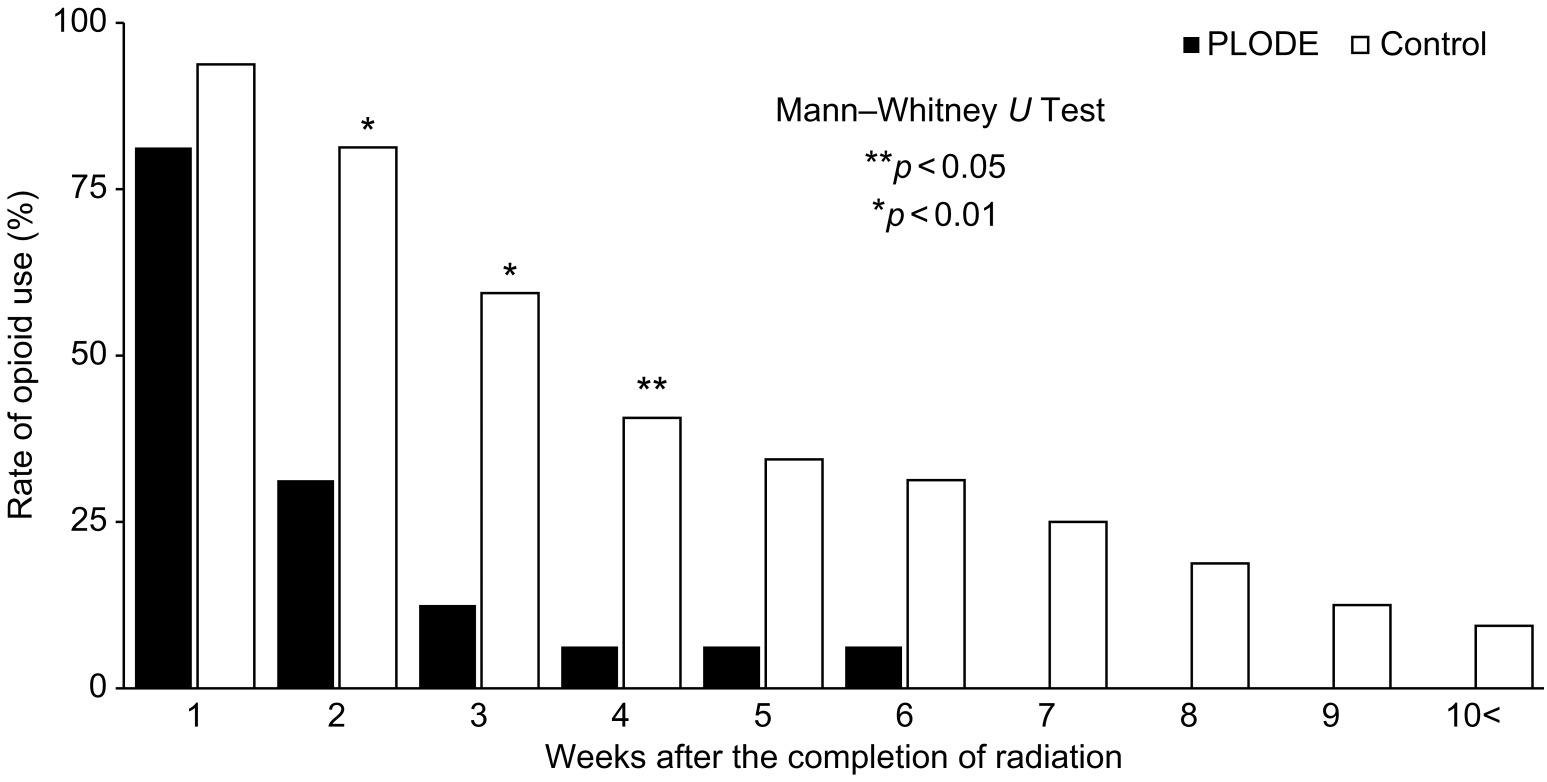
Change in the number of patients using opioid after completion of radiotherapy.

The rate of opioid use two weeks after the completion of radiation was significantly different between the two groups (PLODE vs. control, 31% vs. 81%, *p* < 0.01, **Figure 4**), and only one (6.3%) of the 16 patients in the PLODE group used opioids after four weeks. After five weeks, although there was no significant difference between the two groups, approximately 40% (13/32) of the patients in the control group still used opioids. Altogether, the median duration of opioid use after the completion of radiation was significantly shorter in the PLODE group than in the control (11.5 days [range: 15–50] vs. 24 days [range: 9–46] (**Table 2**). The median duration of opioid use after the completion of radiation due to differences in clinical setting between postoperative and definitive was 7 days [range: 2–20] and 11 days [range: 6–49], respectively, in the PLODE. In the control, the median duration was 28 days [range: 15–36] and 23.5 days [range: 2–153], respectively.

**Table 2:** Duration of opioid use after completion of RT.

	PLODE (n=16)	Control (n=32)	*p*-value
Median duration of opioid use after completion of radiation, days [range]	11.5 [2–49]	24.5 [2–153]	< 0.001
Median total duration of opioid use, days [range]	28 [1–85]	48 [5–177]	< 0.01

The duration of opioid use after completion of RT and the total duration of opioid administration were compared using the Mann-Whitney U test.

We conducted a *post-hoc* power analysis to calculate the *post-hoc* power of our results, which was 77.9%.

### 3.5 Opioid rescue usage after opioid de-escalation by the PLODE program

Among the 16 patients who received an opioid de-escalation in the PLODE program, two patients (12.5%) used rescue doses after the opioid-de-escalation; one patient used a rescue dose twice until the subsequent visit after reducing the dose of an extended-release opioid, and another similarly used it twice after discontinuing an extended-release opioid. None of the patients used a rescue dose before the next outpatient visit.

## 4 Discussion

In patients with locally advanced SCCHN who completed CRT or BRT, a pharmacist-led opioid de-escalation program in collaboration with a physician could reasonably shorten the duration of opioid use for treatment-related pain without apparent exacerbation of pain compared with the historical control group.

Long-term prescription of opioids has potential adverse effects on bodily functions, such as nausea, drowsiness, hypogonadism, gastrointestinal motility disorder, constipation, hyperalgesia, and sleep disorders (17, 18). Therefore, unnecessary opioid use should always be avoided. Further, opioid-induced somnolence, considered one of the signs of a relative overdose of opioids, was observed in 43% of patients even after the completion of CRT in the PLODE program, suggesting the presence of opioid overuse in a fraction of the population. Regarding gastrointestinal side effects, such as nausea, vomiting, and constipation, the preceding two usually disappear within 1–2 weeks; however, opioid-induced constipation (OIC) has minimal or no tolerance and generally increases with the duration of opioid analgesic use. Although the current study could not trace the change in the degree of constipation after tapering or discontinuing opioids, we believe that avoiding unnecessary and relatively excessive use of opioids as revealed by the PLODE program should benefit the patient population, which was experienced by approximately 70% of cases in the group by the time of initiation of the intervention in the current study. Furthermore, our study had a limitation with regards to sample size evaluation. The posterior power of our study was 77.9%, slightly below the desired threshold of 80%. However, it was determined that a certain level of power could still be secured.

Regarding the effect of postoperative or definitive clinical setting on the duration of opioid use, the small sample size in the postoperative group precludes any conclusion, but we do not believe that differences in clinical settings consistently affect the duration of opioid use.

Another issue that should be addressed is pain relapse after de-escalation using the PLODE program. A few patients required opioid rescue doses after opioid de-escalation, while most subjects could successfully taper opioids without re-introduction or re-escalation of the extended-release opioid dose, suggesting the feasibility of the program.

Collaboration between pharmacists and oncologists is essential to ensure safer treatment of patients with cancer. Since the Department of Pharmacy at the National Cancer Center Hospital East (NCCHE) established the first Japanese outpatient clinic where pharmacists worked directly with oncologists in 2007, we have reported the benefits of pharmacists managing side effects in collaboration with oncologists (19–21). Notably, outpatient pharmacists who checked and reviewed both patients’ symptoms and doctors’ prescriptions can directly contribute to the field, as indicated in the current study, which, for the first time, showed the significance of pharmacist-led opioid de-escalation in the setting of radiotherapy in patients with locally advanced SCCHN. Considering the potential correlation between opioid use for managing psychological and spiritual distress and the risk of drug abuse and dependence (22), as well as the strong association between alcohol abuse, commonly observed among HNC patients, and an elevated risk of prolonged opioid abuse (23), we assert that the program supporting the tapering process is highly pertinent and advantageous for the population.

In conclusion, a pharmacist-led opioid de-escalation program is feasible and practical for tapering the drug in patients with SCCHN who require opioids to control radiotherapy-related pain and complete radiotherapy. The program may prevent unnecessary opioid use to avoid jeopardizing toxicities without pain relapse.

## Data availability statement

The original contributions presented in the study are included in the article/supplementary material. Further inquiries can be directed to the corresponding author.

## Ethics statement

This study was approved by the National Cancer Institutional Review Board (approval #2018-362). Written informed consent for participation was not required for this study in accordance with the national legislation and the institutional requirements.

## Author contributions

AH, YU, TF, SS, and MT conceived and designed the study, interpreted the data, and drafted the manuscript. KI, TE, and SO participated in the study concept and design and interpreted the data. MT extracted, managed, and analyzed the data. All authors contributed to the article and approved the submitted version.
